# DEATH AND DYING EDUCATION IN US MEDICAL SCHOOLS: A SCOPING REVIEW

**DOI:** 10.1093/geroni/igad104.1034

**Published:** 2023-12-21

**Authors:** Logan Patterson, Autumn Decker, Raven Weaver, Cory Bolkan, Angelique King, Anna Roman

**Affiliations:** Washington State University, Spokane, Washington, United States; Washington State University, Pullman, Washington, United States; Washington State University, Pullman, Washington, United States; Washington State University Vancouver, Vancouver, Washington, United States; Washington State University, Spokane, Washington, United States; Washington State University, Spokane, Washington, United States

## Abstract

With an aging population, knowledge about death, dying, loss, grief, and end-of-life (EOL) care is an essential skill for most physicians. However, EOL education has been minimally incorporated into medical school curricula in the United States (U.S.); there is a significant need to better prepare medical students for the unique challenges of improving patient wellbeing at end-of-life. To understand how death and dying topics have been incorporated into U.S. medical school education, we conducted a systematic scoping review, using PRISMA guidelines, of published research on death education for medical students between 2010 through 2022. An initial search in EMBASE and PubMed yielded 3,443 citations; 265 articles met the initial inclusion criteria (e.g., research-based, EOL education specific), with 97 articles selected for full-text review. Overall, we found significant variation in the type and content of EOL training in U.S. medical schools. Using a competency-based medical education framework that reflects tangible knowledge, skills, and attitudes as a guide to ensure proper EOL training, a deductive coding approach yielded several themes including: reliance on brief, skills-based workshop interventions and limited longitudinal integration into curricula; general research on student attitudes and wellbeing; and an emphasis on needs assessments highlighting gaps in evidence-based EOL training interventions. Using these findings, we offer applied considerations for teaching EOL related skills in U.S. medical schools. In turn, this work contributes to the enhancement of health across the lifespan and might ultimately lead to a society where a “good death” is not an exception, but a reality.

